# Synthesis and Antibacterial Evaluation of (S,Z)-4-methyl-2-(4-oxo-5-((5-substituted phenylfuran-2-yl) methylene)-2-thioxothiazolidin-3-yl)Pentanoic Acids

**Published:** 2015

**Authors:** Ming-Xia Song, Xian-Qing Deng, Zhi-Yu Wei, Chang-Ji Zheng, Yan Wu, Chang-Shan An, Hu-Ri Piao

**Affiliations:** a *Medical College, Jinggangshan University, Ji'an, Jiangxi, 343009, China. *; b *Yanbian University College of Pharmacy, Yanji, Jilin, 133002, China. *; c *Department of Respiaratory Medicine, Yanbian University Affiliated Hospital, Yanji, Jilin, 133002, China. *

**Keywords:** Furan, Pentanoic acid, Antibacterial activity, Cytotoxicity, MRSA, QRSA

## Abstract

The microbial resistance has become a global hazard with the irrational use of antibiotics. Infection of drug-resistant bacteria seriously threatens human health. Currently, there is an urgent need for the development of novel antimicrobial agents with new mechanisms and lower levels of toxicity. In this paper, a series of (*S,Z*)-4-methyl-2-(4-oxo-5-((5-substitutedphenylfuran-2-yl) methylene)-2-thioxothiazolidin-3-yl)pentanoic acids via a Knoevenagel condensation were synthesized and evaluated for their antibacterial activity *in-vitro*. The synthesized compounds were characterized by IR, ^1^H NMR and MS. The antibacterial test* in-vitro* showed that all of the synthesized compounds had good antibacterial activity against several Gram-positive bacteria (including multidrug-resistant clinical isolates) with minimum inhibitory concentration (MIC) values in the range of 2–4 µg/mL. Especially compounds 4c, 4d, 4e and 4f were the most potent, with MIC values of 2 µg/mL against four multidrug-resistant Gram-positive bacterial strains.

## Introduction

The microbial resistance, caused by the irrational use of antibiotics, has become a global hazard. Infection by drug-resistant bacteria seriously threatens human health. In the case of bacterial resistance growing, looking for antimicrobial agents with new mechanisms becomes an important issue to protect human life, and it has become the concern of the whole society. Therefore, the design and development of new antibacterial drugs have very important significance (1).

**Figure 1:**
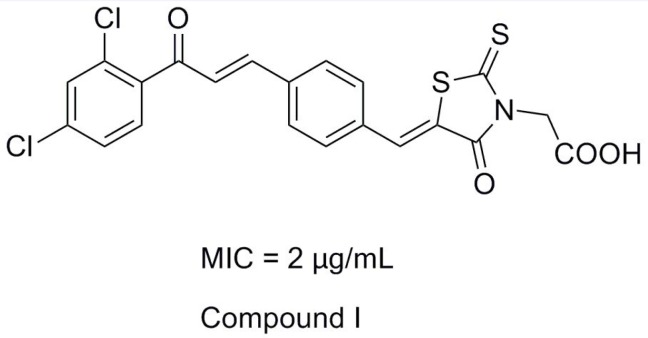
The structure of lead compound (Compound I).

Rhodanine-3-acetic acid and its derivatives bear broad biological activities as anti-epileptic, anti-bacterial, anti-viral and antidiabetic (2-8). For the past few years, our research group devoted ourselves to the study of hybrid compounds of rhodanine-3-acetic acid with some antibacterial fragments (chalcone, parazole or their analogues) (9-16). And we found that rhodanine-N-acetic acid or its analogous 2, 4-thiazolidinedione-N-benzoic acid functionalized with chalcone showed higher levels of antibacterial activity, especially compound I exhibited good inhibition with a MIC value of 2 μg/mL against nine selected Gram-positive strains (including multidrug-resistant clinical isolates) (Figure 1) (14,15). 

**Figure 2:**
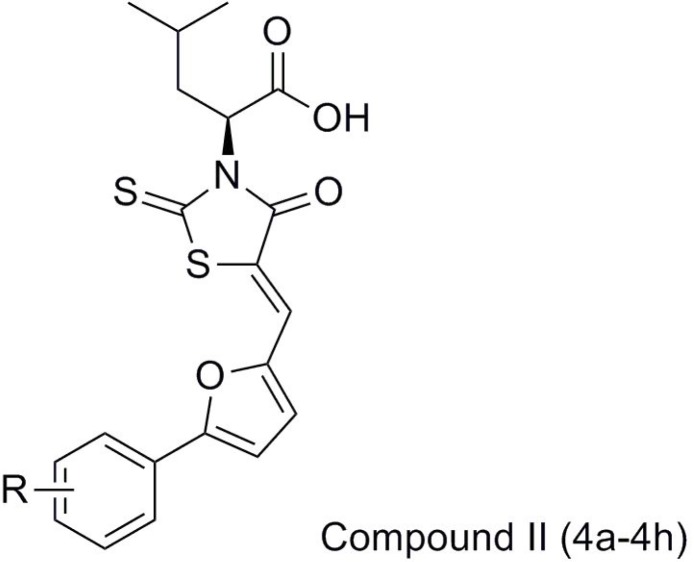
The structure of target compounds (Compound II (4a-4h)).

In the present work, we report the synthesis and antibacterial evaluation of compound II using compound I as the lead compound, which structure-based design mainly focused on the chalcone moiety, was replaced by its isostere 2-phenylfuran and simultaneously introduced by different substituents into the phenyl ring, expecting to get more optimized structures for binding to receptor easily, which consequently could result in their more potent activity. Moreover, the acetic acid group on the 3-position of the rhodanine was also substituted with an aliphatic side chain aiming to increase the bacterial cell wall penetration of these compounds and therefore potentially improve their anti-bacterial activities (Figure 2). 

## Experimental

*Chemistry*

**Figure 3:**
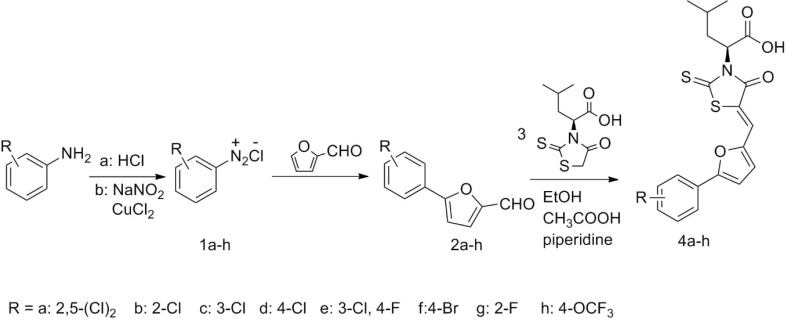
Synthetic route of target compounds (4a-4h).

The target compounds (4a-4h) were synthesized according to the route depicted in Figure 3. Briefly, intermediates 2a-2h were synthesized from substituted aniline by Meerwein arylation reaction using the reported procedure (17). The intermediates 3 were easily prepared from L- leucine according to the reported procedure (18). The target compounds (4a-4h) were obtained via a Knoevenagel condensation of compounds 2a-2h and *(S)*-4-methyl-2-(4-oxo-2-thioxothiazolidin-3-yl) pentanoic acid (3). 

The structures of the desired compounds were confirmed by IR, ^1^H NMR, and high-resolution mass spectroscopy. Taking compound 4b as an example, the high-resolution mass spectrometry of 4b displayed a molecular ion signal at m/z 434.0284, which was corresponding to its theoretical value of 434.0293. In the ^1^H-NMR spectrum of compound 4b, in addition to the aromatic protons of benzene and furan ring protons (δ = 7.50–8.02 ppm), a sharp singlet due to vinylic hydrogen was observed at 7.81 ppm and a broad singlet due to methyne hydrogen linked to nitrogen-atoms was observed at 5.64 ppm. Meanwhile, it has been reported that the (*Z*)- or (*E*)-geometry was readily identified by ^1^H-NMR, as the vinylic proton is more deshielded in the (*Z*)-isomer than the (*E*)-isomer. In (*Z*)-form, vinylic proton appeared at 7.21 ppm due to the magnetic anisotropy effects of carbonyl group on the vinylic proton, while in (*E*)-form the resonance should be around 6.50 ppm (19-20). In our ^1^H-NMR spectra, only a set of signals at 7.81 ppm appeared, which confirmed our products 4 were only in (*Z*)-configuration as thermodynamically favored structures. 

The physicochemical properties of the compounds synthesized are presented in the materials and methods section. Their antibacterial activities were all evaluated by a serial dilution method to obtain the Minimum Inhibitory Concentration (MIC) with different strains including multidrug-resistant clinical isolates.

*Materials and methods*

Melting points were determined in open capillary tubes and are uncorrected. Reaction courses were monitored by TLC on silica gel-precoated F254 Merck plates. Developed plates were examined with Ultra Violet lamps (254 nm). IR spectra were recorded (in KBr) on a FTIR1730 (Perkin Elmer, Massachusetts, USA). ^1^H-NMR spectra were measured on a Bruker AV-300 spectrometer using tetramethylsilane as the internal standard. Mass spectra were measured on a micrOTOF-Q II mass spectrometer (Bruker Daltonik, Bremen, Germany). Specific optical rotation was measured on a Digital automatic polariscope JASCO P-1020 (JASCO, Tokyo, Japan). The major chemicals were purchased from Sigma-Aldrich (St. Louis, MO, USA) and Fluka Companies (Milwaukee, MI, USA). 

*Preparation of compounds*

*5-(* *Substituted-* *phenyl)furan-2-carbaldehyde* *s (* *2a* *-2h)*

A mixture of aniline (30 mmol) and sodium nitrite (30.3 mmol) in hydrochloric acid (8 mL) and water (6 mL) was stirred for 1h at 0 °C. After filtering the reaction, acetone (30 mL), furfural (30 mmol) and cupric chloride (3 mmol) were slowly added to the filtrate, and the mixture was stirred for 12 h at 20 °C. The excess solvent was removed under reduced pressure and the residue was dissolved in ethyl acetate and extracted with water, dried over MgSO4, and the solvent was evaporated under reduced pressure. The resulting residue was purified by silica gel column chromatography (dichloromethane / methanol= 200:1) to obtain a brown solid. 

*(S)-4-methyl-2-(4-oxo-2-thioxothiazolidin-3-yl) pentanoic acid* *(3)*

In a round-bottomed ﬂask equipped with a magnetic stirrer, L-leucine(30.3 mmol) was dissolved with sodium hydroxide (30.3 mmol) in water (25 mL). Then, carbon disulﬁde (30.3 mmol) was added to the reaction mixture, which was stirred vigorously overnight. An aqueous solution of sodium chloroacetate (30.3 mmol) was added and stirring was continued at 23 ?

*(S,Z)-2-(5-((5-(* *substituted-* *phenyl)furan-2-yl)methylene)-4-oxo-2-thioxothiazolidin-3-yl)-4-methylpentanoic acid* *6-( phenyl)thiazolo[3,2-b][1,2,4]triazoles (* *4a* *-4h)*

A mixture of compounds 2a-2h (2.2 mmol), compound 3 (2.0 mmol), 10 drops piperidine and 10 drops glacial acetic acid in ethanol (15 mL) was refluxed for 4 h. After cooling, the solvent was evaporated under reduced pressure. The resulting residue was purified by silica gel column chromatography (dichloromethane / methanol = 100:1) to afford a yellow solid. 

*(S,Z)-2-(5-((5-(2,5-dichlorophenyl)furan-2-yl)methylene)-4-oxo-2-thioxothiazolidin-3-yl)-4-methylpentanoic acid* *(* *4a* *)*

Yield 49%, m.p. 70-72 °C, Mol. weight: 468.99. [*α*]     ：31.2 (c=0.49, CH_3_CO_2_C_2_H_5_). IR (KBr) cm^–1^: 2963 (OH), 1715 (C=O). ^1^H NMR (DMSO-*d*_6_, 300 MHz, ppm): *δ* 0.92 (d, 3H, *J* = 5.8 Hz,CHCH_3_), 0.97 (d, 3H, *J* = 5.4 Hz, CHCH_3_), 1.53 (m, 1H, CH (CH_3_)_2_), 2.05 (m, 1H, CHCH_2_-H_a_), 2.25 (m, 1H, CHCH_2_-H_b_), 5.62 (br.s, 1H, N-CH), 7.49–7.99 (m, 5H, Ar-H), 7.74 (s, 1H, C=CH), 13.41 (s, 1H, COOH). ESI-HRMS calcd for C_20_H_16_C_l2_NO_4_S_2_^-^ ([M-H]^-^): 467.9903; found: 467.9912.

*(S,Z)-2-(5-((5-(2-chlorophenyl)furan-2-yl)methylene)-4-oxo-2-thioxothiazolidin-3-yl)-4-methylpentanoic acid* *(* *4b* *)*

Yield 38%, m.p. 71-73 °C, Mol. weight: 435.04. [*α*]     ：35.4 (c=0.60, CH_3_CO_2_C_2_H_5_). IR (KBr) cm^–1^: 2955 (OH), 1713 (C=O). ^1^H NMR (DMSO-*d*_6_, 300 MHz, ppm): *δ* 0.91 (d, 3H, *J* = 6.6 Hz, CHCH_3_), 0.96 (d, 3H, *J* = 6.4 Hz, CHCH_3_), 1.52 (m, 1H, CH (CH_3_)_2_), 2.05 (m, 1H, CHCH_2_-H_a_), 2.25 (m, 1H, CHCH_2_-H_b_), 5.64 (br.s, 1H, N-CH), 7.50–8.02 (m, 5H, Ar-H), 7.81 (s, 1H, C=CH), 13.41 (s, 1H, COOH). ESI-HRMS calcd for C_20_H_17_ClNO_4_S_2_^-^ ([M-H]^-^): 434.0293; found: 434.0284.

*(S,Z)-2-(5-((5-(3-chlorophenyl)furan-2-yl)methylene)-4-oxo-2-thioxothiazolidin-3-yl)-4-methylpentanoic acid* *(* *4c* *)*

Yield 44%, m.p. 72-74 °C, Mol. weight: 435.04. IR (KBr) cm^–1^: 2969 (OH), 1716 (C=O). ^1^H NMR (DMSO-*d*_6_, 300 MHz, ppm): *δ* 0.93 (d, 3H, *J* = 6.1 Hz, CHCH_3_), 0.96 (d, 3H, *J* = 6.4 Hz, CHCH_3_), 1.52 (m, 1H, CH (CH_3_)_2_), 2.06 (m, 1H, CHCH_2_-H_a_), 2.24 (m, 1H, CHCH_2_-H_b_), 5.65 (br.s, 1H, N-CH), 7.48–7.99 (m, 5H, Ar-H), 7.78 (s, 1H, C=CH), 13.41 (s, 1H, COOH). ESI-HRMS calcd for C_20_H_17_ClNO_4_S_2_^-^ ([M-H]^-^): 434.0293; found: 434.0292.

*(S,Z)-2-(5-((5-(4-chlorophenyl)furan-2-yl)methylene)-4-oxo-2-thioxothiazolidin-3-yl)-4-methylpentanoic acid* *(* *4d* *)*

Yield 46%, m.p. 76-78 °C, Mol. weight: 435.04. IR (KBr) cm^–1^: 2955 (OH), 1713 (C=O). ^1^H NMR (DMSO-*d*_6_, 300 MHz, ppm): *δ* 0.91 (d, 3H, *J* = 5.9 Hz, CHCH_3_), 0.96 (d, 3H, *J* = 6.1 Hz, CHCH_3_), 1.53 (m, 1H, CH (CH_3_)_2_), 2.05 (m, 1H, CHCH_2_-H_a_), 2.23 (m, 1H, CHCH_2_-H_b_), 5.64 (br.s, 1H, N-CH), 7.47–7.95 (m, 5H, Ar-H), 7.77 (s, 1H, C=CH), 13.39 (s, 1H, COOH). ESI-HRMS calcd for C_20_H_18_ClNNaO_4_S_2_^+^ ([M+Na]^+^): 458.0258; found: 458.0256.

*(S,Z)-2-(5-((5-(3-chloro-4-fluorophenyl)furan-2-yl)methylene)-4-oxo-2-thioxothiazolidin-3-yl)-4-methylpentanoic acid* *(4e)*

Yield 35%, m.p. 75-77 °C, Mol. weight: 453.03. [*α*]     ：36.9 (c=0.60, CH_3_CO_2_C_2_H_5_). IR (KBr) cm^–1^: 2975 (OH), 1711 (C=O). ^1^H NMR (DMSO-*d*_6_, 300 MHz, ppm): *δ* 0.91 (d, 3H, *J* = 6.4 Hz, CHCH_3_), 0.96 (d, 3H, *J* = 6.4 Hz, CHCH_3_), 1.52 (m, 1H, CH (CH_3_)_2_), 2.05 (m, 1H, CHCH_2_-H_a_), 2.26 (m, 1H, CHCH_2_-H_b_), 5.80 (br.s, 1H, N-CH), 7.04–8.15 (m, 5H, Ar-H), 7.74 (s, 1H, C=CH), 13.39 (s, 1H, COOH). ESI-HRMS calcd for C_20_H_16_ClFNO_4_S_2_^-^ ([M-H]^-^): 452.0199; found: 452.0189.

*(S,Z)-2-(5-((5-(4-bromophenyl)furan-2-yl)methylene)-4-oxo-2-thioxothiazolidin-3-y*

*l)-4-methylpentanoic acid* *(4f)*

Yield 39%, m.p. 83-85 °C, Mol. weight: 478.99. [*α*]     ：32.9 (c=0.64, CH_3_CO_2_C_2_H_5_). IR (KBr) cm^–1^: 2964 (OH), 1711 (C=O). ^1^H NMR (DMSO-*d*_6_, 300 MHz, ppm): *δ* 0.91 (d, 3H, *J* = 6.6 Hz, CHCH_3_), 0.96 (d, 3H, *J* = 6.5 Hz, CHCH_3_), 1.52 (m, 1H, CH (CH_3_)_2_), 2.05 (m, 1H, CHCH_2_-H_a_), 2.24 (m, 1H, CHCH_2_-H_b_), 5.63 (br.s, 1H, N-CH), 7.47–7.85 (m, 5H, Ar-H), 7.77 (s, 1H, C=CH), 13.38 (s, 1H, COOH). ESI-HRMS calcd for C_20_H_17_BrNO_4_S_2_^-^ ([M-H]^-^): 477.9788; found: 477.9784.

*(S,Z)-2-(5-((5-(2-fluorophenyl)furan-2-yl)methylene)-4-oxo-2-thioxothiazolidin-3-yl)-4-methylpentanoic acid* *(* *4g* *)*

Yield 42%, m.p. 74-76 °C, Mol. weight: 419.07. [*α*]     ：46.1 (c=0.31, CH_3_CO_2_C_2_H_5_). IR (KBr) cm^–1^: 2973 (OH), 1712 (C=O). ^1^H NMR (DMSO-*d*_6_, 300 MHz, ppm): *δ* 0.91 (d, 3H, *J* = 6.6 Hz, CHCH_3_), 0.96 (d, 3H, *J* = 6.5 Hz, CHCH_3_), 1.55 (m, 1H, CH (CH_3_)_2_), 2.06 (m, 1H, CHCH_2_-H_a_), 2.25 (m, 1H, CHCH_2_-H_b_), 5.66 (br.s, 1H, N-CH), 7.25–7.97 (m, 5H, Ar-H), 7.81 (s, 1H, C=CH), 13.40 (s, 1H, COOH). ESI-HRMS calcd for C_20_H_18_FNNaO_4_S_2_^+^ ([M+Na]^+^): 442.0553; found: 442.0558.

*(S,Z)-4-methyl-2-(4-oxo-2-thioxo-5-((5-(4-(trifluoromethoxy)phenyl)furan-2-yl)met*

*hylene)thiazolidin-3-yl)pentanoic acid* *(* *4h* *)*

Yield 48%, m.p. 79-81 °C, Mol. weight: 485.06. -[*α*]     ：30.7 (c=0.63, CH_3_CO_2_C_2_H_5_). IR (KBr) cm^–1^: 2969 (OH), 1715 (C=O). ^1^H NMR (DMSO-*d*_6_, 300 MHz, ppm): *δ* 0.91 (d, 3H, *J* = 5.9 Hz, CHCH_3_), 0.97 (d, 3H, *J* = 6.1 Hz, CHCH_3_), 1.53 (m, 1H, CH (CH_3_)_2_), 2.06 (m, 1H, CHCH_2_-H_a_), 2.25 (m, 1H, CHCH_2_-H_b_), 5.64 (br.s, 1H, N-CH), 7.48–8.05 (m, 5H, Ar-H), 7.78 (s, 1H, C=CH), 13.37 (s, 1H, COOH). ESI-HRMS calcd for C_21_H_17_F_3_NO_5_S_2_^-^ ([M-H]^-^): 484.0506; found: 484.0510.

*Pharmacology*

*Evaluation of anti-bacterial activity in-vitro*

The *in-vitro* anti-bacterial activity was evaluated using a 96-well microtiter plate and a serial dilution method to obtain the Minimum Inhibitory Concentration (MIC) with different strains including multidrug-resistant clinical isolates. Oxacillin, norfloxacin, gatifloxacin and moxifloxacin were used as positive controls. 

The micro-organisms used in the present study were *S. aureus* (*S. aureus* *RN *4220, *S. aureus KCTC *209, *S. aureus KCTC *503), and *Escherichia coli* (*E. coli* 1356). The strains of multidrug-resistant clinical isolates were methicillin-resistant *Staphylococcus aureus* (*MRSA CCARM* 3167 and *MRSA CCARM* 3506) and quinolone-**resistant** *Staphylococcus aureus* (*QRSA CCARM* 3505 and *QRSA CCARM* 3519). Clinical isolates were collected from various patients hospitalized in several clinics.

Test bacteria were grown to mid-log phase in Mueller-Hinton broth (MHB) and diluted 1000-fold in the same medium. The bacteria of 10^5^ CFU/mL were inoculated into MHB and dispensed at 0.2 mL/well in a 96-well microtiter plate. As positive controls, oxacillin and norfloxacin were used. Test compounds were prepared in DMSO, the final concentration of which did not exceed 0.05%. A two-fold serial dilution technique (21) was used to obtain final concentrations of 64-0.5 μg/mL. The MIC was defined as the concentration of a test compound that completely inhibited bacteria growth during 24 h incubation at 37 °C. Bacteria growth was determined by measuring the absorption at 650 nm using a microtiter enzyme-linked immunosorbent assay (ELISA) reader. All experiments were carried out three times.

*Evaluation of* *cytotoxicity* *in-vitro*

Human cervical (Hela) cell monolayers were used as an in vitro model of cervicovaginal epithelium for testing the cytotoxicity of the new compounds. Hela cells were grown in Dulbecco modified Eagle medium supplemented with fetal bovine serum (10%), and antibiotics (penicillin-streptomycin mixture [100 U/mL]). Cells at 80 to 90% confluence were split by trypsin (0.25% in PBS; pH 7.4), and the medium was changed at 24 h intervals. The cells were cultured at 37 °C in a 5% CO_2_ incubator. The cells were grown to 3 passages and approximately 1×10^4 ^cells were seeded into each well of a 96-well plate and allowed to incubate overnight to allow cells to attach to the substrate. After 24 h, the medium was replaced with DMEM supplemented with 10% FBS containing various concentrations of test compounds and incubated for 48 h. Then 10 µL of MTT solution (5 mg/mL in PBS) was added to each well. After incubation for 4 h, the medium was removed and the resulting formazan crystals were dissolved with 100 µL DMSO. After shaking 10 min, the optical density was measured at 570 nm using a microtiter ELISA reader. The assay was conducted four times. The IC_50_ values were defined as the concentrations inhibiting 50% of cell growth.

## Results and Discussion

*Evaluation of anti-bacterial activity in-vitro*

The results of compounds 4a-4h against three Gram-positive strains (*S. aureus RN* 4220, *S. aureus KCTC* 209 and *S. aureus KCTC* 503) and one Gram-negative strain (*E. coli *1356) are shown in Table 1. And the results of compounds against several clinical isolates of multidrug-resistant Gram-positive bacterial strains (methicillin-resistant *Staphylococcus aureus* (*MRSA CCARM* 3167 and *MRSA CCARM* 3506) and quinolone-**resistant** *Staphylococcus aureus* (*QRSA CCARM* 3505 and *QRSA CCARM* 3519)) are shown in Table 2.

As shown in Table 1, compounds 4a-4g exhibited good inhibitory activity against* S. aureus RN* 4220 with MICs of 2 µg/mL, which had comparable activity to the positive control norfloxacin (MIC = 2 µg/mL), but weaker than the positive control oxacillin (MIC = 1 µg/mL), gatifloxacin and moxifloxacin (MIC = 0.25 µg/mL). Compounds 4a-4h displayed good activity against *S. aureus KCTC* 503 with MIC values of 4 µg/mL. However, these compounds displayed moderate to weak potency against *S. aureus KCTC* 209 with MIC values ranging from 8-64 µg/mL. None of the compounds showed any inhibitory activity against the Gram-negative strain *E. coli *1356 at 64 µg/mL. 

**Table 1:** Inhibitory activity (MIC, µg/mL) of compounds 4a-4h against bacteria.

**Compound**	**Gram-positive ** **strains**			**Gram-negative** **strains**
	***S. aureus***			***E. coli***
	**4220** a	**209** b	**503** c	**1356** d
4a	2	>64	4	>64
4b	2	32	4	>64
4c	2	32	4	>64
4d	2	16	4	>64
4e	2	16	4	>64
4f	2	8	4	>64
4g	2	64	4	>64
4h	4	16	4	>64
Norfloxacin	2	2	2	16
Oxacillin	1	1	1	>64
Gatifloxacin	0.25	2	4	16
Moxifloxacin	0.25	2	2	>64

a *S. aureus* RN 4220, *Staphylococcus aureus* RN 4220

b *S. aureus* 503, *Staphylococcus aureus* 503

c *S. aureus* 209, *Staphylococcus aureus* 209

d *E. coli* 1356, *Escherichia coli* CCARM 1356

As shown in Table 2, against the four multidrug-resistant Gram-positive bacterial strains, compounds 4a-4h presented good activity with MIC values of 2-8 µg/mL. Among them, compounds 4c, 4d, 4e and 4f were the most potent, with MIC values of 2 µg/mL, comparable to the leading compound I (MIC = 2 µg/mL). Compounds 4c, 4d, 4e and 4f were four or two-fold more potent than norfloxacin (MIC = 8 µg/mL and 4 µg/mL), 32-fold more active than oxacillin (MIC > 64 µg/mL), comparable or slightly weaker than gatifloxacin and moxifloxacin (MIC = 1 or 2 µg/mL) against *MRSA CCARM *3167 and 3506 strains. And they were 32-fold more potent than norfloxacin (MIC > 64 µg/mL) and 4 or 2-fold gatifloxacin and moxifloxacin (MIC = 8 or 4 µg/mL), but slightly weaker activity to oxacillin (MIC = 1 µg/mL) against the QRSA CCARM 3505 and 3519 strains. 

**Table 2:** Inhibitory activity (MIC, µg/mL) of compounds 4a-4h against clinical isolates of multidrug-resistant Gram-positive strains.

**Compound**	**Multidrug-resistant ** **Gram-positive** ** strains**			
	***MRSA***		***QRSA***	
	**3167** a	**3506** b	**3505** c	**3519** d
4a	2	8	4	2
4b	2	2	2	4
4c	2	2	2	2
4d	2	2	2	2
4e	2	2	2	2
4f	2	2	2	2
4g	2	2	2	4
4h	4	4	4	4
Norfloxacin	8	4	>64	>64
Oxacillin	>64	>64	1	1
Gatifloxacin	2	1	8	4
Moxifloxacin	1	1	4	4

a MRSA 3167, methicillin-resistant *S. aureus *CCARM 3167

b MRSA 3506, methicillin-resistant *S. aureus *CCARM 3506

c QRSA 3505, quinoline-resistant *S. aureus *CCARM 3505

d QRSA 3519, quinoline-resistant *S. aureus *CCARM 3519

*Evaluation of cytotoxicity in-vitro*

To see whether the antibacterial activity of compound 4a is selectively toxic to bacteria, its cytotoxicity was evaluated (Table 3). As shown in the Table 3, the IC_50_ (12.41 µg/mL) of compound 4a is higher than its MIC values indicating that compound 4a is selectively toxic to bacteria. 

**Table 3:** Cytotoxic activity of compound 4a against HeLa cell.

**Compound**	**IC**_50_ ** (** **µg/mL** **)** a
4a	12.41

a IC_50_ is the concentrations required to inhibit 50% of cell growth.

## Conclusion

A series of (*S,Z*)-2-(5-((5-(substituted-phenyl)furan-2-yl)methylene)-4-oxo-2-thioxothiazolidin-3-yl)-4-methylpentanoic acid (4a-4h) were synthesized and evaluated for antibacterial activity. All of the compounds showed good antibacterial activities against multidrug-resistant strains of clinical isolates. Compounds 4c, 4d, 4e and 4f showed the most potent levels of activity (MIC = 2 µg/mL) against selected MRSA and QRSA strains. Cytotoxicity evaluation suggested that the promising antibacterial activity of these compounds is not attributed to their toxicity. While the mechanism of action of this compound remains unknown, efforts to establish the cause of their antibacterial activity are ongoing and will be reported in due course.
